# Tales of significance

**DOI:** 10.1186/s12915-016-0275-0

**Published:** 2016-06-23

**Authors:** Graham Bell

**Affiliations:** BMC Biology, BioMed Central, 236 Gray’s Inn Road, London, WC1X 8HB UK

## Abstract

In this experiment, the authors were interested in testing the effect of a small molecule inhibitor on the ratio of males and females in the offspring of their model Dipteran species. The authors report that in a wild-type population, ~50 % of offspring are male. They then test the effect of treating females with the chemical, which they think might affect the male:female ratio compared with the untreated group. They claim that there is a statistically significant increase in the percentage of males produced and conclude that the drug affects sex ratios.

## Commentary

**Fig. 1.**
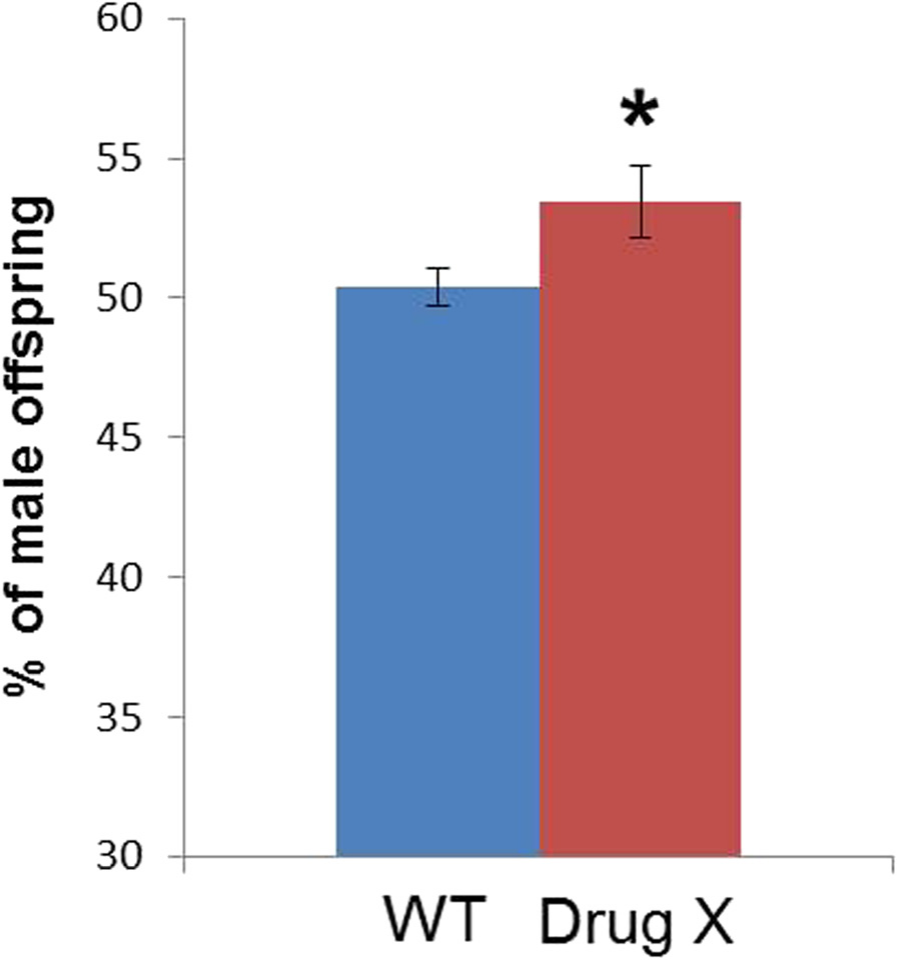
The percentage of male offspring produced by untreated (*WT*) flies or female flies treated with drug X. *P < 0.05, one-tailed t-test, error bars show SD

Previous examples in this series have drawn attention to some problems with *p* values and statistical significance. Choosing the right test to use to analyse data is another area of possible confusion. In this case, the conclusion that the drug causes a statistically significant difference is not supported by the data because the authors used an inappropriate statistical test in their analysis. Their hypothesis was that there would be a change in the ratio of the sexes, but in either direction—either more males or fewer males. In that case, a two-tailed test is needed. However, the two-tailed test did not reach statistical significance. The authors then used a one-tailed test in order to test the hypothesis that the drug increased the percentage of males born; this gave a *p* value of <0.05, which the authors indicate in the work (Fig. 1).

A one-tailed test is used to determine if there is a difference in the means in one direction only (more males; or fewer males; but not either outcome); because of this, one-tailed *p* values are half of the two-tailed value in most statistical tests and reach statistical significance faster than two-tailed counterparts. Though there is nothing wrong with using a one-tailed test in principle—if there is a good reason to assume the difference in means would be in one direction only—the authors erred in their initial choice and also should not change the test post hoc.

